# SV2A PET imaging in human neurodegenerative diseases

**DOI:** 10.3389/fnagi.2024.1380561

**Published:** 2024-04-18

**Authors:** Mahsa Shanaki Bavarsad, Lea T. Grinberg

**Affiliations:** Memory and Aging Center, Weill Institute for Neurosciences, University of California, San Francisco (UCSF), San Francisco, CA, United States

**Keywords:** synaptic vesicle glycoprotein 2A (SV2A), synaptic loss, SV2A PET, 11C-UCB-J, neurodegeneration

## Abstract

This manuscript presents a thorough review of synaptic vesicle glycoprotein 2A (SV2A) as a biomarker for synaptic integrity using Positron Emission Tomography (PET) in neurodegenerative diseases. Synaptic pathology, characterized by synaptic loss, has been linked to various brain diseases. Therefore, there is a need for a minimally invasive approach to measuring synaptic density in living human patients. Several radiotracers targeting synaptic vesicle protein 2A (SV2A) have been created and effectively adapted for use in human subjects through PET scans. SV2A is an integral glycoprotein found in the membranes of synaptic vesicles in all synaptic terminals and is widely distributed throughout the brain. The review delves into the development of SV2A-specific PET radiotracers, highlighting their advancements and limitations in neurodegenerative diseases. Among these tracers, 11C-UCB-J is the most used so far. We summarize and discuss an increasing body of research that compares measurements of synaptic density using SV2A PET with other established indicators of neurodegenerative diseases, including cognitive performance and radiological findings, thus providing a comprehensive analysis of SV2A’s effectiveness and reliability as a diagnostic tool in contrast to traditional markers. Although the literature overall suggests the promise of SV2A as a diagnostic and therapeutic monitoring tool, uncertainties persist regarding the superiority of SV2A as a biomarker compared to other available markers. The review also underscores the paucity of studies characterizing SV2A distribution and loss in human brain tissue from patients with neurodegenerative diseases, emphasizing the need to generate quantitative neuropathological maps of SV2A density in cases with neurodegenerative diseases to fully harness the potential of SV2A PET imaging in clinical settings. We conclude by outlining future research directions, stressing the importance of integrating SV2A PET imaging with other biomarkers and clinical assessments and the need for longitudinal studies to track SV2A changes throughout neurodegenerative disease progression, which could lead to breakthroughs in early diagnosis and the evaluation of new treatments.

## 1 Introduction

### 1.1 Synaptic loss in neurodegenerative conditions

A range of neurological disorders features synaptic abnormalities (Counts et al., 2006; Scheff et al., 2007). Research based on biopsy and postmortem examinations in patients with moderate to severe AD pathology underscores that synapse loss is a strong predictor of cognitive decline (DeKosky and Scheff, 1990; Terry et al., 1991). Until recently, the sole method for measuring synaptic density was through direct examination of brain tissue. The presynaptic vesicle protein synaptophysin (SYP) has been utilized as the “gold standard” marker for assessing synaptic integrity in most studies, given that SYP is abundant in synaptic vesicles (Takamori et al., 2006; Clare et al., 2010).

PET imaging employs probes linked to radiopharmaceuticals to quantify receptors, transporters, enzymes, and proteins *in vivo*. PET tracers for detecting misfolded proteins associated with neurodegenerative conditions such as β-amyloid and tau in the brain are extensively used in research settings, with some receiving clinical approval. However, the distribution of tau and β-amyloid offers limited predictive value for determining cognitive decline in AD and are not useful for other neurodegenerative conditions (Leuzy et al., 2019; Bullich et al., 2021; Groot et al., 2022; Pemberton et al., 2022).

To improve clinical prognosis for neurodegenerative diseases, new PET tracers have been designed in the past years to quantify synaptic density. This review focuses on PET tracers to detect synaptic vesicle glycoprotein 2 (SV2) protein family, with a particular emphasis on SV2A, as a tool in neurodegenerative diseases including Alzheimer’s disease (AD), frontotemporal dementia (FTD), Parkinson’s disease (PD), dementia with Lewy bodies (DLB), Huntington’s disease (HD), progressive supranuclear palsy/Richardson syndrome (PSP), and corticobasal syndrome (CBD). We discuss the evidence of the utility of SV2A PET tracers as tools for monitoring disease progression and predicting clinical outcomes and contrast the results with the evidence available of changes in density detected by direct examination of human brains.

### 1.2 Synaptic vesicle glycoprotein 2A (SV2A) and its role in synaptic function

The SV2 protein family belongs to the group keratan sulfate proteoglycans (Scranton et al., 1993). SV2 plays a pivotal role in regulating vesicle secretion in both neurons and endocrine cells (Buckley and Kelly, 1985; Portela-Gomes et al., 2000). The significant conservation of SV2 across eukaryotic species underscores its importance in governing vesicular dynamics (Bindra et al., 1993). Most studies on SV2 localization in the brain were performed in rats and a comprehensive mapping in the human brain has yet to be accomplished. Among the three SV2 isoforms, SV2A stands out as the predominant variant, exhibiting widespread expression in all gray matter structures. On the other hand, SV2B is found in neocortical regions and the hippocampus, and SV2C is exclusively localized to subcortical regions such as the striatum, pallidum, midbrain, brainstem, substantia nigra, and olfactory bulb (Bajjalieh et al., 1994; Varnäs et al., 2020; Pazarlar et al., 2022). SV2A regulates presynaptic calcium levels by transporting calcium into synaptic vesicles (Mendoza-Torreblanca et al., 2013). When SV2A is dysfunctional, presynaptic Ca2^+^ accumulates, triggering abnormal neurotransmitters and destabilizing the synaptic system (Janz et al., 1999). The exact role of SV2A in synaptic vesicle cycling and neurotransmitter release is not fully understood. It is hypothesized that SV2A plays a part in transporting neurotransmitters within synaptic vesicles, modulating exocytosis, and fine-tuning synaptic function (Mendoza-Torreblanca et al., 2013). Furthermore, SV2A protein is believed to play an important role in synaptogenesis by interacting with other presynaptic proteins leading to the formation of new synapses (Chang and Südhof, 2009). SV2A is consistently and uniformly found in synaptic vesicle terminals throughout the brain (Bajjalieh et al., 1994), rendering it a compelling candidate for identifying and quantifying synaptic vesicles across various brain regions. Additionally, the appeal of SV2A as a marker of synaptic density is further enhanced by the minimal variability in the number of SV2A copies per synaptic vesicle (Mutch et al., 2011).

### 1.3 Development of SV2A PET radiotracers for *in vivo* measuring of synaptic density

In the past few years, several PET tracers were developed to visualize SV2A in the human brain. Levetiracetam, an antiepileptic drug, binds specifically to SV2A. However, the initial radioligand, 11C-levetiracetam (Cai et al., 2014), was abandoned due to its low binding affinity (Danish et al., 2017). Next, three specific ligands were developed, also derived from the common pharmacophore of levetiracetam but with improved binding affinity characteristics as potential radiotracers for SV2A PET imaging: 11C-UCB-A (4-(3,5-Difluorophenyl)-1-((1-methyl-1H-imidazol-5-yl)methyl)pyrrolidin-2-one), 18F-UCB-H (1-((3-Fluoropyridin-4-yl)methyl)-4-(3,4,5-trifluorophenyl)pyrrolidin-2-one), and 11C-UCB-J ((R)-1-((3-(11C-methyl-11C)pyridin-4-yl)methyl)-4-(3,4,5-trifluorophenyl)pyrrolidin-2-one).

Among them, 11C-UCB-J displayed the most favorable imaging characteristics and pharmacokinetics when evaluated in non-human primates. It exhibited high brain uptake, rapid and reversible binding kinetics, high specific binding signals (measured by non-displaceable binding potential - BP_ND_ values) in various brain regions, low non-specific binding in white matter, and a relatively fast metabolism rate in the plasma. Higher SV2A binding was observed mainly in gray matter brain regions associated with SV2A expression (Nabulsi et al., 2016). Conversely, 11C-UCB-A uptake rate in nonhuman primate brains was slow, making the quantification analysis more complicated (Estrada et al., 2016) and 18F-UCB-H showed lower specific binding signals in nonhuman primate brains (Nabulsi et al., 2016) due to lower SV2A binding affinity (Mercier et al., 2014). When tested in humans, these three SV2A ligands yield similar results to non-human primate studies. Whereas 11C-UCB-A and 18F-UCB-H showed similar limitations as before (Bahri et al., 2017; Lubberink et al., 2017), 11C-UCB-J demonstrated more promising imaging properties, high specific binding signals, and good test–retest reproducibility (Finnema et al., 2016, 2018). A regional mapping autoradiography study with the radioligand 11C-UCB-J was conducted in both non-human primate and human brain tissue. The study unveiled specific binding of SV2A in all gray matter structures, demonstrating a consistent regional expression pattern of SV2A across both species’ brains (Varnäs et al., 2020). In 2016, Finnema and his colleagues compared SV2A density in baboons identifying a good linear correlation between the volume distribution of 11C-UCB-J binding measured by PET and the regional SV2A distribution determined by Western blot analysis and homogenate binding assay obtained in postmortem brain in all gray matter regions. The study also demonstrated a strong correlation between regional synaptic density of SV2A and synaptophysin, the gold standard marker of synaptic integrity, in Western blot analysis, with R^2^ values of 0.92 for all regions and 0.90 for gray matter regions. This suggests that SV2A could be a promising alternative marker for quantifying synaptic density and may serve as a potential therapeutic target (Finnema et al., 2016).

The first human 11C-UCB-J PET study was conducted in individuals with epilepsy (Finnema et al., 2016). Further studies defined the centrum semiovale, a white matter region, as a reference region for 11C-UCB-J PET analyses (Mertens et al., 2020; Rossano et al., 2020). However, subsequent studies suggested the cerebellum as a better reference region because SV2A binding in the centrum semiovale shows too much signal variation in normal controls (Mecca et al., 2020).

Although 11C-UCB-J is a promising radiotracer for targeting SV2A, the short half-life of carbon-11 (20 min) limits its production and applicability. To overcome this limitation, versions of UCB-J labeled with Fluorine-18 (half-life =110 min) have been developed. Currently, there are different 18F-labeled SV2A tracers: 18F-SynVesT-1 (named as 18F-MNI-1126 and 18F-SDM-8), 18F-SynVesT-2 (18F-SDM-2) and 18F-SDM-16. In human PET studies, 18F-SynVesT-1 demonstrated similar pharmacokinetic characteristics but higher binding potential (BP_ND_) values than 11C-UCB-J (Li et al., 2021; Naganawa et al., 2021). On the other hand, 18F-SynVesT-2 exhibited a lightly lower binding potential (BP_ND_) but had faster pharmacokinetics in comparison to 11C-UCB-J in nonhuman primates (Cai et al., 2017). A recent nonhuman PET study with 18F-SDM-16 showed the highest SV2A uptake affinity and metabolic stability compared to other available SV2A PET radioligands (Constantinescu et al., 2019; Zheng et al., 2022). Assessment of newly developed 18F-labeled SV2A PET imaging probes in human studies is ongoing. Table 1 shows the summarized human SV2A PET studies in neurodegenerative disorders, and the association between SV2A PET synaptic density and cognitive performance in human studies of neurodegenerative disorders is shown in Table 2.

**Table 1:** List of SV2A PET studies of neurodegenerative disorders in humans.

Disease	Year	Study population	Participants (n)		Radiotracer	Outcome measure	Reference region	Main outcomes	Ref
			Patients	Controls					
AD	2018	aMCI/AD	10	11	11C-UCB-J	BP_ND_	Centrum semiovale	Lower binding (41%) in the hippocampus of AD.	Chen et al. (2018)
AD	2020	aMCI/AD	34	19	11C-UCB-J	DVR	Cerebellum	Widespread reduction 11C-UCB-J binding in medial temporal and neocortical regions in AD.	Mecca et al. (2020)
AD	2020	MCI/AD	24	19	18F-UCB-H	Vt	Centrum semiovale Cerebellum	Lower binding mainly in the hippocampus (31%), cortical areas (11–18%) and thalamus (16%) in AD.	Bastin et al. (2020)
AD	2020	aMCI	10	10	11C-UCB-J 18F-MK-6240	SUVR	Centrum semiovale	Lower 11C-UCB-J binding negatively associated with higher 18F-MK 6240 uptake in the medial temporal lobe in AD.	Vanhaute et al. (2020)
AD	2021	aMCI/AD	14	11	11C-UCB-J 18F-FDG	DVR	Cerebellum	Similar decrease in 11C-UCB-J and 18F-FDG uptake in the medial temporal lobe of AD, with less magnitude of the reduction of 11C-UCB-J binding in neocortical regions in comparison to 18F-FDG.	Chen et al. (2021)
AD	2021	AD	7	-	11C-UCB-J 18F-flortaucipir	BP_ND_	Centrum semiovale	Lower 11C-UCB-J binding associated with higher reginal 18F-flortaucipir uptake in AD.	Coomans et al. (2021)
AD	2021	aMCI/AD	38	19	11C-UCB-J 11C-PiB	DVR	Cerebellum	Global amyloid deposition inversely associated with hippocampal SV2A binding in aMCI individuals but not those with mild dementia.	O'Dell et al. (2021)
AD	2022	aMCI/AD	45	19	11C-UCB-J	DVR	Cerebellum	Positive association between synaptic density and cognitive performance in AD.	Mecca et al. (2022b)
AD	2022	aMCI/AD	10	10	11C-UCB-J 18F-flortaucipir	DVR	Cerebellum	Inverse association between entorhinal tau deposition and hippocampal synaptic density in AD.	Mecca et al. (2022a)
AD	2022	AD	12	16	11C-UCB-J	DVR	Centrum semiovale	Lower 11C-UCB-J binding (range from 2 to 25%), with the greatest reduction in the caudate (25%), hippocampus (24%) and thalamus (19%) in AD.	Venkataraman et al. (2022)
AD	2023	AD	24	19	18F-UCB-H	Vt	-	Lower binding in the bilateral hippocampus and amygdala in AD. Additionally, regardless of different changes in myelin, iron, and synapse degeneration, the most affected areas are the left and right hippocampus, amygdala, right pallidum, left fusiform and temporal lobe in AD.	Moallemian et al. (2023)
AD	2023	aMCI/AD	64	30	18F-SynVesT-1	SUVR	Cerebellum	Lower 18F-SynVesT-1 density in bilateral cortex and hippocampus in the dementia group but not in MCI.	Zhang et al. (2023)
AD	2023	aMCI/AD	33	17	11C-UCB-J	DVR	Cerebellum	Hippocampal synaptic density inversely associated with hippocampal mean diffusivity measured by DTI in AD.	Silva-Rudberg et al. (2024)
PD	2020	PD	12	12	11C-UCB-J	BP_ND_	Centrum semiovale	Lower binding in substantia nigra (45%), red nucleus (31%) and locus coeruleus (17%) in PD.	Matuskey et al. (2020)
PD	2020	Early drug-naive PD	12	16	11C-UCB-J	Vt	Centrum semiovale	Lower binding in the caudate (15.1%), thalamus (11.6%), putamen (9.7%), and substantia nigra (6.9%) in PD patients.	Wilson et al. (2020)
PD	2020	PD	30	20	11C-UCB-J	BP_ND_	Centrum semiovale	Lower binding in substantia nigra (15.2%) of PD.	Delva et al. (2020)
PD	2021	PD	21	15	11C-UCB-J	SUVR-1	Centrum semiovale	Lower binding in the substantia nigra of PD.	Andersen et al. (2021)
PD	2023	PD	21	15	11C-UCB-J 18F-FDG	SUVR-1	Centrum semiovale	Lower binding in PD; reduction ranged from (1%) in the cerebellum to (18%) in the substantia nigra, and regional differences for cerebral glucose uptake ranged from (1%) in the posterior cingulate cortex to (20%) in the insular cortex.	Andersen et al. (2023)
DLB	2021	DLB/PDD	13	15	11C-UCB-J	SUVR-1	Centrum semiovale	Widespread lower binding in multiple cortical regions in DLB/PDD.	Andersen et al. (2021)
DLB	2020	DLB	2	10	11C-UCB-J	BP_ND_	Centrum semiovale	Lower binding in thalamus, parieto-occipital, middle and superior frontal cortices in DLB.	Nicastro et al. (2020)
DLB	2023	DLB/PDD	13	15	11C-UCB-J 18F-FDG	SUVR-1	Centrum semiovale	Lower binding in DLB/PDD, with the largest reduction in the insular cortex (46%) and a higher decline in FDG uptake in the occipital cortex (48%).	Andersen et al. (2023)
FTLD	2021	bvFTD	12	12	18F-UCB-H	Vt	Ubiquitous distribution of 18F-UCB-H in the brain did not allow the identification of a “reference region”	18F-UCB-H uptake tended to decrease (41%) in the right parahippocampal gyrus in bvFTD.	Salmon et al. (2021)
FTLD	2023	bvFTD	11	25	11C-UCB-J	BP_ND_	Centrum semiovale	Lower binding bilaterally in the medial and dorsolateral frontal regions, inferior frontal gyri, anterior and posterior cingulate gyri, insular cortex, and medial temporal lobe in bvFTD.	Malpetti et al. (2023)
FTLD	2021	(3 presymptomatic C9orf72 carriers +1 bvFTD symptomatic patient)	4	19	11C-UCB-J	BP_ND_	Centrum semiovale	Lower binding in the thalamus in carriers and decreased 11C-UCB-J uptake in the left frontotemporal cortex and subcortical regions in the bvFTD patient.	Malpetti et al. (2021)
ALS	2022	ALS	21	26	18F-SynVesT-1	SUVR	Centrum semiovale	Lower uptake in right temporal lobe and the bilateral inferior frontal gyrus, anterior cingulate, and hippocampus–insula region. No correlation between SUVR and clinical scores.	Tang et al. (2022)
PSP CBS	2020	PSP CBS	29	15	11C-UCB-J	BP_ND_	Centrum semiovale	Lower binding (up to 50%) in cortical and subcortical regions in PSP and CBS.	Holland et al. (2020)
PSP CBD	2021	PSP CBD	36	27	11C-UCB-J	BP_ND_	Centrum semiovale	Lower binding across the cortex and subcortical regions in both PSP and CBD, in addition 11C-UCB-J measuer is more sensetive than atrophy.	Mak et al. (2021)
PSP CBD	2022	PSP CBD	35	19	11C-UCB-J 18F-flortaucipir	BP_ND_	Centrum semiovale	Negative correlation between 11C-UCB-J binding and 18F-flortaucipir uptake among brain regions of both PSP and CBD.	Holland et al. (2022)
PSP CBD	2023	PSP CBD	22	33	11C-UCB-J	BP_ND_	Centrum semiovale	Annual lower binding in frontal lobe (3.5%) and in the right caudate (3.9%) in both PSP and CBD.	Holland et al. (2023)
HD	2022	HD (premanifest and manifest early-stage)	18	15	11C-UCB-J	BP_ND_	Centrum semiovale	Lower binding in putamen (33%), caudate (31%), pallidum (30%) and whole gray matter (12%) in manifest early-stage, while lower synaptic uptake in putamen (19%) and caudate (16%) in premanifest group.	Delva et al. (2022)
HD	2023	HD (premanifest and manifest early-stage)	17	13	11C-UCB-J	SUVR-1	Centrum semiovale	Longitudinal lower 11C-UCB-J binding in the frontal and parietal cortex in the premanifest, but no changes in early manifest group.	Delva et al. (2023)

AD, Alzheimer disease; ALS, amyotrophic lateral sclerosis; aMCI, amnestic mild cognitive impairment; BPND, binding potential (specific binding); bvFTD, behavioral variant frontotemporal dementia; CBD, Corticobasal degeneration; DLB, dementia with Lewy bodies; DVR, distribution volume ratio; FTLD, Frontotemporal lobar degeneration; HD, Huntington disease; PD, Parkinson disease; PDD, PD with dementia; PiB, Pittsburgh compound B; MCI, mild cognitive impairment; PSP, Progressive supranuclear palsy; SUVR-1, standardized uptake value ratio; VT, volume of distribution.

**Table 2:** Association between SV2A PET synaptic density and cognitive performance in human studies of neurodegenerative disorders.

Disease	Clinical manifestations	Ref
AD	Low hippocampal synaptic density associated with higher Clinical Dementia Rating scale Sum of Boxes (CRD-SB) and lower episodic memory scores in AD.	Mecca et al. (2020) and Chen et al. (2018)
AD	Low hippocampal synaptic density correlated with cognitive decline and unawareness of memory problems in AD.	Vanhaute et al. (2020) and Bastin et al. (2020)
AD	Low synaptic density associated with lower global cognition and specific cognitive domain scores (language, executive function, processing speed, and visuospatial ability) in AD.	Mecca et al. (2022b)
AD	Low synaptic density in the hippocampus and parietal lobe associated with impaired language (picture naming and semantic fluency) in AD.	Venkataraman et al. (2022)
AD	Low synaptic density in right insular cortex and bilateral caudal middle frontal gyrus correlated with cognitive decline in AD.	Zhang et al. (2023)
PD	Low synaptic density in the brain stem correlated with motor severity scores and total disease burden in PD.	Wilson et al. (2020)
DLB	Low synaptic density in the middle frontal gyrus correlated with cognitive impairment in executive function of DLB/PDD.	Andersen et al. (2021)
FTLD	Low synaptic density in caudate nucleus and the anteromedial prefrontal cortex correlated with greater anosognosia for clinical symptoms in bvFTD.	Salmon et al. (2021)
FTLD	Low synaptic density in frontal and cingulate regions correlated with cognitive impairments in bvFTD.	Malpetti et al. (2023)
PSP CBS	Low synaptic density in cortical and subcortical regions correlated with disease severity in PSP and CBS.	Holland et al. (2020)
PSP CBS	Low synaptic density in the frontal lobe correlated with disease progression and cognitive state in both PSP and CBD.	Holland et al. (2023)
HD	Low synaptic density in putamen correlated with motor and cognitive impairment in HD.	Delva et al. (2022)

AD, Alzheimer disease; bvFTD, behavioral variant frontotemporal dementia; CBD, Corticobasal degeneration; CDR-SB, Clinical Dementia Rating scale Sum of Boxes; DLB, dementia with Lewy bodies; FTLD, Frontotemporal lobar degeneration; HD, Huntington disease; PD, Parkinson disease; PDD, Parkinson disease with dementia; PSP, Progressive supranuclear palsy.

## 2 SV2A PET in neurodegenerative diseases

### 2.1 SV2A PET in Alzheimer’s disease (AD)

AD is the most studied neurodegenerative disorder associated with synaptic dysfunctions. It is generally accepted that synaptic loss is the best correlate of cognitive decline and parallels plaque and neurofibrillary tangle (NFT) deposition, making changes in synaptic density an attractive biomarker (Overk and Masliah, 2014; Robinson et al., 2014; de Wilde et al., 2016; Colom-Cadena et al., 2020). Very little is known about changes in SV2A density in AD brain tissue. In an autoradiography study comparing AD with non-demented subjects, Metaxas et al. (2019) showed no differences in the frontal cortical binding density of 3H-UCB-J between groups. They also observed the negative correlation between 3H-UCB-J binding level and AT8 immunoreactivity (phosphorylated tau marker) in the AD group (Metaxas et al., 2019).

A recent autoradiography study by Mikkelsen et al. (2023) reported a significant negative correlation between 3H-UCB-J and age. Furthermore, they found lower 3H-UCB-J binding levels in the middle frontal gyrus (but not in the parietal, temporal, or occipital cortex) of individuals with AD compared to age-matched controls (Mikkelsen et al., 2023).

In contrast, multiple studies (albeit the majority conducted by the same group leading to sample overlapping) using 11C-UCB-J PET imaging in patients positive for AD biomarkers reveal decreased binding in several cortical areas. In one of the first studies by Chen et al. from Yale University, contrasting 10 AD (amyloid PET positive) patients at MCI and mild dementia stages and 11 (amyloid PET negative) controls, 11C-UCB-J binding in hippocampus was reduced by 41% in the AD group and the change remained significant even after partial volume correction. Hippocampal 11C-UCB-J binding correlated with episodic memory scores. Of note, the change in magnitude in 11C-UCB-J binding was greater than hippocampal volume loss (22%) (Chen et al., 2018). In a follow-up study with a larger study population (34 AD and 19 controls), the cerebellum was used as the reference region instead of the centrum semiovale, as the former showed less signal variability across subjects. This time, in addition to the hippocampus, other areas including entorhinal cortex, amygdala, and neocortical regions showed statistical differences between AD and controls (Mecca et al., 2020). These results suggest a potential of 11C-UCB-J PET as a biomarker to measure regional brain integrity in AD. The next logical step would be to compare 11C-UCB-J PET with other commonly used neuroimaging markers. One of these studies (14 AD vs. 11 controls) showed that 11C-UCB-J PET binding in neocortical areas was less pronounced than the changes detected through 18F-FDG (Fludeoxyglucose) PET (a tracer measuring glucose metabolism, a proxy of neuronal activity, commonly used in AD workup) (Minoshima et al., 2021), except for the temporal areas, where the changes were similar in magnitude (Chen et al., 2021). Among the explanations for this relative lack of cortical SV2A density changes may include the fact that synaptic loss is more linked to tau than amyloid pathology (Blennow et al., 1996) and tau pathology only spreads to the neocortex at later AD stages (Hyman et al., 2012). Two additional investigations, both conducted by the same research team at Yale University, aimed to provide insights into this issue. In the first (38 AD (amyloid PET positive) cases −14 at aMCI and 24 at mild dementia stages), comparing 11C-PiB PET uptake (measure of Aβ deposition) and 11C-UCB-J PET binding, the investigators did not find any same region correlation between the two tracers (O'Dell et al., 2021). Conversely, the second small study (10 AD (5 at aMCI and 5 at mild dementioa stages) and 10 controls) using 11C-UCB-J PET and 18F-flortaucipir (tau) PET suggested a possible inverse correlation between both tracers, including a stronger inverse correlation between tau deposits in the entorhinal cortex and SV2A density in the hippocampus (Mecca et al., 2022a). Another study with 10 MCI vs. 10 controls similarly identified that higher 18F-MK-6240(Tau PET) binding is inversely related to lower 11CUCB-J binding (*p* = 0.02, *r* = −0.76) in medial temporal lob (temporal, mesotemporal, and hippocampal regions) (Vanhaute et al., 2020). A third study with 7 amyloid positive individuals found a moderate to strong negative correlation between flortaucipir and 11C-UCB-J PET, but only in the four subjects with moderate to high flortaucipir signal (combined 12 cortical ROIs), whereas no or positive correlations were found in individuals with low flortaucipir binding (Coomans et al., 2021). Moreover, alterations in tau PET exhibited a stronger correlation with cognitive performance than changes observed in 11C-UCB-J PET (Vanhaute et al., 2020). The most recent study by the Yale University group compared changes in 11C-UCB-J PET and mean diffusivity measured by DTI and showed an inversed correlation of both, but only in the AD group, not in controls (Silva-Rudberg et al., 2024). Still on comparing SV2A density with other image-based measures of brain integrity, Venkataraman et al. compared longitudinal changes in 11C-UCB-J PET with PET tracers designed to detect mitochondrial function or cell stress in eight amyloid-positive participants. Although the study detected 11C-UCB-J PET changes in caudate, thalamus and hippocampus (areas with high synaptic density on baseline) these changes did not correlate with blood flow changes. Conversely, mitochondrial marker 18F-BCPP-EF DVR_CS-1_ (CS: centrum semiovale as a reference region) for mitochondrial complex I (MC1) showed more significant changes over time (Venkataraman et al., 2022). An additional crucial aspect for understanding the utility of 11C-UCB-J PET as a biomarker in AD continuum is to examine how changes in 11C-UCB-J PET correlate with clinical outcomes in comparison to other biomarkers. In a cross-sectional study, the same group from Yale University found a low to moderate significant correlation between 11C-UCB-J binding (a composite value of eight ROIs affected in AD) and scores of five different cognitive domains, even after partial volume correlation (Mecca et al., 2022b). Global cognition scores showed the stronger association with 11C-UCB-J PET and this correlation was more robust than brain volume and cognition. Overall, alterations in 11C-UCB-J PET appear to serve as an early indicator of brain dysfunction in AD compared to volume loss. Nevertheless, uncertainties persist regarding whether SV2A is a superior biomarker than other available markers. More extensive longitudinal multi-marker studies in well-characterized cohorts are necessary to answer these questions. A group from the University of Liege in Belgium published two studies employing 18F-UCB-H PET in 24 amyloid-PET positive subjects and 19 controls. The first study found reduced uptake in several cortical areas and thalamus. The hippocampus showed the highest magnitude of changes and hippocampal changes were associated with cognitive decline and an unawareness of memory (Bastin et al., 2020). The follow up study examined the concurrence of synaptic density measurements using 18F-UCB-H PET with changes in iron content, myelination, brain volume using neuroimaging techniques. As in studies comparing different techniques against 11C-UCB-J PET, the areas showing changes diverge among the markers. The authors suggest that combining multimodal imaging in multivariate analysis is the best strategy to reduce noise (Moallemian et al., 2023). In fact, a big limitation of implementing new imaging techniques in the clinical practice is the lack of validation against ground truth histology making it challenging to dissociate signal from noise. Finally, in the only study using 18F-SynVesT-1 PET (corrected for partial volume effect and using cerebellum as the reference) to evaluate synaptic alterations in AD to date (64 amyloid-positive - 33 at dementia and 31 at MCI stage vs. 30 controls), authors combined functional MRI and cognitive measuring to explore the correlations between SV2A density changes and markers of brain integrity. Changes in SV2A uptakes were identified in several brain areas in the dementia group but not in MCI Such changes had a low but significant correlation with MMSE scores, which means results show weak effect size but significant (right insular cortex R = 0.213 *p* = 0.038, right middle frontal gyrus R = 0.278 *p* = 0.037, and left middle frontal gyrus R = 0.124 *p* = 0.039). However, fMRI with seeds in middle frontal cortices and insula pointed to strong correlations between changes in connectivity and synaptic density in dementia and MCI groups corroborating postmortem studies that suggest a link between synaptic integrity and functional connectivity (Zhang et al., 2023).

### 2.2 SV2A PET in Lewy body disease the neuropathological counterpart of Parkinson’s disease (PD) and dementia with Lewy bodies (DLB)

Lewy body disease (LBD) is an age-related neurodegenerative condition with deposits of alpha-synuclein in subcortical and cortical neurons. It manifests clinically as Parkinson’s disease (PD), Parkinson’s disease dementia (PDD), or Dementia with Lewy bodies (DLB) (Myslinski et al., 1989; Kon et al., 2020). There is clear evidence indicating the importance of synaptic alterations, including glutamatergic synaptic transmission, in the pathogenesis of LBD (Schulz-Schaeffer, 2010; Villalba and Smith, 2013; Colom-Cadena et al., 2017). Therefore, measures of SV2A density are a potentially attractive biomarker in LBD. Once again, there are almost no studies investigating SV2A density in the brain tissue of LBD cases. One study performed qualitative analysis in the substantia nigra and striatum of 7 LBD cases, 2 PSP, 7 AD, and 7 healthy controls immunostained for SV2A and SV2C (the latter almost exclusively found in subcortical nuclei). The qualitative analysis found no changes in SV2A in any disease groups compared to controls. Nevertheless, in substantia nigra and putamen of LBD cases presenting as PD, researchers observed SV2C signal appearing as puncta instead of a diffuse signal. This pattern was also noted to a lesser extent in cases presenting as DLB, with no observable changes in other disease groups. This study, along with others, established an association between SV2C and LBD, underscoring the need for the development of a PET tracer targeting SV2C. Another investigation by Matuskey et al. applied 3H-UCB-J autoradiography on substantia nigra and putamen in 15 subjects with moderate and severe LBD stages and 13 controls and found a 17% decrease in SV2A density in substantia nigra. Notably, within the same study, the authors employed 11C-UCB-J PET imaging to evaluate 12 PD patients and 12 controls. The results demonstrated significant reductions in 11C-UCB-J uptake in subcortical regions, including the substantia nigra (45%), red nucleus (31%), and locus coeruleus (17%) (Matuskey et al., 2020). Although 11C-UCB-J PET seems promising in this study, it is concerning that the magnitude of PET changes was almost 3 times greater than the autoradiography changes, which could suggest that the tracer is not specific in *in vivo* studies. In a comparison of 12 drug-naïve, early PD subjects, and 16 healthy controls, Wilson et al. reported a significant reduction in 11C-UCB-J volume of distribution in the caudate (15.1%), thalamus (11.6%), putamen (9.7%), substantia nigra (6.9%), and cortical regions of PD patients, but no changes in the volume of distribution of 11C-SA-4503 (sigma 1 receptor) or 18F-BCPP-EF (mitochondrial complex 1). Moreover, lower brain stem 11C-UCB-J binding was negatively associated with Movement Disorder Society Unified Parkinson’s Disease Rating Scale part III (MDS-UPDRS part III) and Hoehn and Yahr (H&Y) scale scores (Wilson et al., 2020). Another study with 30 PD subjects at early clinical stages and 20 controls demonstrated a significant reduction (15.2%) in 11C-UCB-J binding in the substantia nigra of PD patients, but not in the putamen, whereas 18F- FE-PE2I PET binding was decreased in the putamen only. Although the nigral 11C-UCB-J uptake and putamen 18F-FE-PE2I uptake showed correlation, none of these metrics correlated with any clinical scores (Delva et al., 2020). A few studies were performed on individuals with DLB. Nicastro et al. used 11C-UCB-J PET in two DLB patients and 10 controls. To control for AD pathology, a common comorbid finding in LBD expressing as DLB, the subjects also underwent PET imaging of amyloid (11C-PiB) and tau (18F-AV-1451) (Nicastro et al., 2020). Compared to controls, both DLB subjects showed reduced binding potential in several areas, most prominent in thalamus, parieto-occipital, middle and superior frontal cortices. Notably, subcortical areas involved in LBD such as midbrain and putamen showed no differences between cases and control. Also, there was no correlation between 11C-UCB-J BP_ND_ and volume loss. In another cohort, Anderson et al., performed 11C-UCB-J PET, 18F-FDG PET, and volumetry based on T1 MRI and neuropsychological testing in 21 subjects with PD lacking cognitive decline, 13 subjects with PD dementia or DLB and 15 controls. SUVR1 values for 11C-UCB-J PET was decreased in the substantia nigra only in the PD-lacking dementia subjects, but also in other cortical areas in PDD/DLB group. The SUVR1 decrease matched well with volumetric changes and the SUVR1 levels in frontal cortex correlated with cognitive function (Andersen et al., 2021). However, in a follow-up study by the same groups, changes in 18F-FDG exceeded those observed in 11C-UCB-J PET (Andersen et al., 2023). This discrepancy could imply that both changes represent distinct facets of the pathogenesis, rendering 11C-UCB-J PET a complementary tool in the evaluation of LBD. Alternatively, it raises the possibility that 11C-UCB-J PET might be less advantageous compared to the presently employed biomarker. A challenge in DLB and PDD is that a significant percentage of these individuals have comorbid AD pathology, which can also affect SV2A density and 18F-FDG PET. Fine mapping of SV2A density in brain tissue of LBD subjects, a better understanding of what 11C-UCB-J measures *in vivo*, and longitudinal studies are needed to properly evaluate the utility of SV2A tracers for LBD.

### 2.3 SV2A PET in bvFTD, ALS and C9orf72 carriers

Behavioral variant frontotemporal lobar degeneration (bvFTD) features progressive deterioration of personality, social comportment, and cognition. bvFTD is a common cause of dementia in individuals under the age of 65 (Rascovsky et al., 2011). The molecular signature of bvFTD is heterogeneous, including TDP-43 or tau inclusions, and its diagnosis still requires postmortem examination (Perry et al., 2017). Regardless of molecular signature, there are limited postmortem studies on synaptic changes in bvFTD cases, none specifically for the SV2 family (Lipton et al., 2001; Clare et al., 2010). Likewise, very few published studies have examined the utility of SV2A PET tracers in bvFTD. In a study using 18F-UCB-H in 12 patients with probable bvFTD, 12 healthy controls, and 12 AD, 18F-UCB-H uptake tended to decrease in the right anterior parahippocampal gyrus in bvFTD, although no reductions were observed in frontal regions, even when the medial prefrontal cortex displayed the most significant degree of atrophy. There were no differences in tracer binding between bvFTD and AD (Salmon et al., 2021). In a recent study, Malpetti et al. used 11C-UCB-J to assess 11 patients with probable bvFTD and 25 healthy controls and detected a prominent decline in binding bilaterally in the regions most expected to be affected in bvFTD, i.e., frontal regions, cingulate cortex, insula and medial temporal lobe. Furthermore, there was a significant correlation between cognitive dysfunction and decreased 11C-UCB-J uptake in the frontal and cingulate regions (Malpetti et al., 2023). Unfortunately, it is challenging to draw conclusions based on two studies with contradictory results (but also different tracers). One possible explanation is that the initial study (Salmon et al., 2021) combined ventral- and dorsal-predominant bvFTD cases, introducing a confounding factor in the results. Alternatively, it could be suggested that the tracer 18F-UCB-H is less effective or specific in binding to SV2A compared to 11C-UCB-J, a possibility corroborated by the literature (Constantinescu et al., 2019). The better utility of 11C-UCB-J to detect changes in FTLD spectrum is corroborated by another paper, also by Malpetti et al. in which reduction in 11C-UCB-J uptake was detected in the thalamus of three presymptomatic C9orf72 carriers (Malpetti et al., 2021). C9orf72 carriers usually develop bvFTD, amyotrophic lateral sclerosis or both, and the thalamus is one of the earliest affected areas (Lee et al., 2017). In any case, histological studies mapping SV2A density in brain tissue in FTLD spectrum disorders and studies comparing tracer binding and histological studies in the same brains for validation are critically warranted to enable interpreting PET studies results.

A recent synaptic PET study using 18F-SynVesT-1 on 21 amyotrophic lateral sclerosis (ALS) patients and 26 heatlhy controls from China (Tang et al., 2022) revealed lower uptake in the ALS group compared to controls in specific brain regions including the right temporal lobe, bilateral inferior frontal gyrus, anterior cingulate, and hippocampus–insula area. This study also observed reduced uptake in the bilateral superior temporal gyrus, hippocampus–insula, anterior cingulate, and left inferior frontal gyrus in ALS patients with cognitive impairment versus controls. The molecular characteristics of these cases remain unidentified. Pathologically, ALS primarily affects motor neurons, except in cases of ALS-FTD or ALS with concurrent neuropathologies like AD. Although 6 of the patients had cognitive decline (of unknown origin), the study identified cortical changes in SV2A PET uptake in patients without cognitive decline, indicating the need for further research to understand what this tracer measure.

### 2.4 SV2A PET in primary tauopathies

Progressive supranuclear palsy (PSP) and corticobasal degeneration (CBD) are primary four-repeat tauopathies featuring parkinsonism and cognitive deficits (Kovacs, 2017). The few studies examining synaptic density in these two conditions suggest a decrease in synaptophysin density, although less prominent than in AD and with unclear clinical correlations (Bigio et al., 2001; Lipton et al., 2001). Our search retrieved four studies using 11C-UCB-J PET in patients likely with PSP and CBD, all conducted by the same Cambridge University group, thus presenting a potential overlap of cases. The first study evaluated presumed synaptic loss in 14 patients with Richardson’s syndrome (usually caused by PSP pathology), 15 patients with corticobasal syndrome (9 of whom were negative for beta-amyloid, thus presumably with CBD or PSP pathology), and 15 healthy controls. 11C-UCB-J binding was reduced up to 50% in atrophic cortical areas. Additionally, 11C-UCB-J binding was associated with clinical scores (Holland et al., 2020). A follow-up study with 22 Richardson’s syndrome and 14 amyloid-negative CBD compared 11C-UCB-J PET and MRI-based Orientation Dispersion Imaging (ODI – a measure of dendritic arborization). Both measures were greater in magnitude than atrophy and correlated in disease-associated areas, potentially suggesting they are more sensitive than atrophy (Mak et al., 2021). A significant positive correlation was observed between 11C-UCB-J and the Addenbrooke’s Cognitive Examination -Revised, ACE-R total score (*R* = 0.52; *p* = 0.01). There was a significant negative correlation between 11C-UCB-J binding and the PSP (*R* = −0.61; *p* < 0.01) and CBD (*R* = −0.72; *p* < 0.001) rating scales (Mak et al., 2021). Another follow-up, cross-sectional study compared 11C-UCB-J PET to flortaucipir (tau) PET, showing a moderate negative correlation between the tracers that became weaker in patients with more advanced disease stages. The authors intended to test the hypothesis that regions with higher synapse density at baseline accumulate more tau across time, but they acknowledge that it would require a longitudinal study (Holland et al., 2022).

A step in this direction was published in 2023 by showing results of longitudinal 11C-UCB-J PET (about 16 month interval) with 22 PSP or CBD (amyloid-negative). The study found longitudinal changes in 11C-UCB-J PET binding basal ganglia in addition to cerebellum and parietofrontal cortex in CBD and frontal, cingulate cortices and midbrain in PSP, areas affected by these diseases. The magnitude of longitudinal changes correlated well with symptom progression (Holland et al., 2023). These studies show promise regarding the potential utility of 11C-UCB-J PET for detecting early brain changes and monitor disease progression in PSP and CBD, two entities with poor choices of *in vivo* biomarkers. However, it is essential to understand what are expected SV2A changes in these conditions based on direct brain tissue examination. Moreover, studies are needed to validate these findings.

### 2.5 SV2A PET in Huntington’s disease (HD)

HD is an autosomal dominant disorder caused by trinucleotide repeat expansion in the *HTT gene,* leading to neurodegeneration with clinical cognitive, motor, and behavioral deficits (Budworth and McMurray, 2013). Although a full understanding of synaptic dysfunction in HD remains unknown, the literature shows changes in the density of various synaptic proteins in HD and suggests a link between synaptic density and clinical symptoms (Smith et al., 2007; Smith-Dijak et al., 2019; Huang et al., 2020; Martín-Flores et al., 2020). For instance, the density of synapse-associated protein 97 (SAP97) and postsynaptic density protein-95 (PSD-95) is increased in the hippocampus, while PSD-95 levels are decreased in the striatum (Fourie et al., 2014). An older study using autoradiography clearly demonstrated a decrease in striatal levels of several vesicular neurotransmitter transporters, although SV2A was not measured, in HD brains (Suzuki et al., 2001). Thus, synaptic PET tracers such as those targeting SV2A have the potential to offer a non-invasive measure of disease burden. Regarding studies on SV2A density in tissue of HD patients, a recent study compared the frontal cortex of one HD and one healthy control case (both free of AD pathology) using 3H-UCB-J autoradiography and SV2A immunofluorescence (Bertoglio et al., 2022). This pilot study shows a decrease in SV2A in HD.

A recent PET study using 11C-UCB-J and 18F-FDG in 7 premanifest and 11 early manifest HD mutation carriers vs. 15 healthy controls found a decrease in 11C-UCB-J uptake in the striatum of premanifest cases that extended to pallidum, cerebellum, and cortical areas in early manifest cases, whereas 18F-FDG PET only showed changes in the striatum in both groups. Also, 11C-UCB-J uptake in putamen was correlated with motor and cognitive impairment in both HD groups (Delva et al., 2022). The same group reported on a follow-up longitudinal study with the same cohort in which repeated measures were obtained approximately 21 months after the baseline. Although the premanifest group showed lower 11C-UCB-J PET binding in frontal and parietal cortex in the second scan compared to controls (vs. only basal ganglia changes on baseline), longitudinal changes were not apparent after multiple corrections. Despite a clear clinical decline and brain volume loss, the early manifest group had no changes in 11C-UCB-J PET values compared to baseline, and the only area showing longitudinal 18F-FDG-PET change was the occipital cortex (Delva et al., 2023). Altogether, this suggests that 11C-UCB-J PET may be more sensitive to early brain changes in HD compared to the traditionally used 18F-FDG PET, but the value of 11C-UCB-J PET in measuring disease spread still requires further investigation with larger cohorts.

## 3 Conclusions and future directions

This manuscript has explored the advancements in SV2A PET imaging for neurodegenerative diseases, with a specific focus on 11C-UCB-J, the most used tracer to date. A growing number of different SV2A radiolabeled probes are being used in clinical studies, and the number of studies focusing on neurodegenerative conditions has grown exponentially in the past few years (Tables 1, 2). Many studies originate from a few groups, leading to a limited and non-diverse subject pool. Conversely, these groups have consistently proposed methodological improvements in each new paper, benefiting the entire field.The main questions these pioneering studies interrogated and their limitations were related to the issues listed below:

1. Alignment of changes detected by SV2A PET with changes detected by currently used biomarkers and neuropathological progress of the diseases. There are inconsistent results when SV2A PET, especially using 11C-UCB-J is compared to other image-based modalities. Recent studies using the cerebellum as a reference region show more alignment of results than earlier studies using the centrum semiovale as a reference. A recurrent issue is the misalignment between brain areas showing the greatest changes in SV2A PET and those identified through MRI-based volumetry. The lack of studies mapping SV2A distribution in human brains at several stages of neurodegenerative conditions and lack of studies validating SV2A PET imaging against the histological ground truth in humans persist as important limitations for the interpretation of SV2A PET imaging in clinical studies. It is unclear whether decreases in SV2A binding reflect a loss of neurons, decreased density of vesicles expressing SV2A, and whether off-target signal contributes to the results.
2. Comparison with 18F-FDG PET: 18F-FDG PET is the most commonly used imaging study to identify areas of reduced metabolic activity in the brain. The few studies comparing 18F-FDG PET and 11C-UCB-J suggest that 18F-FDG PET detects greater magnitude of changes than the latter. This raises questions about the sensitivity and specificity of 11C-UCB-J PET. It is suggested that 11C-UCB-J is less influenced by confounding factors, indicating that these two modalities may highlight different aspects of the pathogenic processes. More extensive studies including comparisons with clinical outcomes and a better understanding of the neurobiological basis of SV2A PET signal are needed to understand the differences and utility of SV2A PET compared to 18F-FDG PET.
3. Correlation with Clinical Decline: the existing studies provide mixed results regarding the correlation of SV2A PET changes with clinical outcomes. Nearly all research including clinical to SV2A Pet correlations demonstrate a level of correlation. Nonetheless, the nature of this correlation varies across studies examining the same condition. This variation could suggest diversity among participants or differences in tracer types, reference areas, and outcome metrics. Consequently, there is a need for larger, longitudinal studies with standardized methodologies to accurately identify the clinical significance of these tracers. A deeper understanding of what these tracers measure in the human brain is essential for future research.

Overall, SV2A PET imaging shows promise in detecting changes in presynaptic density and correlating with clinical decline, offering an insightful measure of brain integrity, but some challenges and limitations persist.

Future research should focus on generating quantitative neuropathological maps of SV2A density in neurodegenerative diseases. This will aid in interpreting SV2A PET results and enhance our understanding of synaptic changes in these conditions. Additionally, expanding sample sizes and diversity in clinical studies will provide more robust and generalizable findings. Integrating SV2A PET imaging with other diagnostic tools and clinical outcomes will be pivotal in advancing the diagnosis and monitoring of neurodegenerative diseases.

## Author contributions

MB: Conceptualization, Data curation, Investigation, Methodology, Writing – original draft, Writing – review & editing. LG: Conceptualization, Data curation, Funding acquisition, Methodology, Project administration, Supervision, Writing – original draft, Writing – review & editing.
